# A systematic review of ankle fracture-dislocations: Recent update and future prospects

**DOI:** 10.3389/fsurg.2022.965814

**Published:** 2022-08-09

**Authors:** Mu-Min Cao, Yuan-Wei Zhang, Sheng-Ye Hu, Yun-Feng Rui

**Affiliations:** ^1^ Department of Orthopaedics, Zhongda Hospital, School of Medicine, Southeast University, Nanjing, Jiangsu, China; ^2^ School of Medicine, Southeast University, Nanjing, Jiangsu, China; ^3^ Orthopaedic Trauma Institute (OTI), Southeast University, Nanjing, Jiangsu, China; ^4^ Trauma Center, Zhongda Hospital, School of Medicine, Southeast University, Nanjing, Jiangsu, China

**Keywords:** ankle fracture-dislocations, injury mechanism, complications, functional outcomes, management

## Abstract

**Background:**

Ankle fracture-dislocations are one of the most severe types of ankle injuries. Compared to the simple ankle fractures, ankle fracture-dislocations are usually more severely traumatized and can cause worse functional outcomes. The purpose of this study was to review the previous literatures to understand the anatomy, mechanisms, treatment, and functional outcomes associated with ankle fracture-dislocations.

**Methods:**

The available literatures from January 1985 to December 2021 in three main medical databases were searched and analyzed. The detailed information was extracted for each article, such as researchers, age, gender, groups, type of study, type of center research, level of evidence, significant findings, study aim, cause of injury, time from injury to surgery, type of fracture, direction of dislocation, follow-up, postoperative complications and functional evaluation scores.

**Results:**

A total of 15 studies (1,089 patients) met the inclusion criteria. Only one study was a prospective randomized trial. The top-ranked cause of injury was high-energy injury (21.3%). Moreover, the most frequent type of fracture in ankle dislocations was supination-external rotation (SER) ankle fracture (43.8%), while the most common directions of dislocation were lateral (50%) and posterior (38.9%).

**Conclusions:**

Collectively, most ankle fracture-dislocations are caused by high-energy injuries and usually have poor functional outcomes. The mechanism of injury can be dissected by the ankle anatomy and Lauge-Hansen's classification. The treatment of ankle fracture-dislocations still requires more detailed and rational solutions due to the urgency of occurrence, the severity of injury, and the postoperative complications.

## Introduction

Ankle fractures are one of the most widespread fractures in adults, with a morbidity of 174 per 100,000 per years (1). The dislocation of ankle fracture is a more severe injury and is characterized by the loss of alignment of tibial and talar articular surfaces. The characteristics and mechanisms of pure ankle dislocations have been summarized in a previous systematic review (2). However, the progress of research related to the ankle fracture-dislocations have not been well summarized and analyzed recently.

The patients who experience the ankle fracture-dislocations tend to have poorer functional outcomes (3–6). This may be due to the fact that higher-violence injuries exacerbate the damage to ligaments surrounding the ankle joint. Hence, the treatment of this type of injury also needs to be supported by a higher level of evidence to develop a more detailed and better treatment process, including the emergency management, surgical timing, surgical approach and so on. The purpose of this study was to provide a systematic review of the epidemiology, anatomy, mechanism of injury, treatment and prognosis of the ankle fracture-dislocations, so as to provide certain reference for the relevant researches in the future.

## Methods

### Search strategy

The electronic literatures were searched from PubMed, Web of science and Scopus databases using the search terms of “ankle fracture-dislocations”, “dislocated ankle fractures”, “fracture-dislocation of the ankles” and “Malleolar fracture dislocation”. The overall search procedures, including literatures search, data extraction and quality evaluation were performed by the two independent reviewers. The referenced papers included in reviewed articles were also examined, and the final search was performed on 5 January 2022.

### Study selection

Studies were excluded if they were case reports, case series, conference abstracts. Moreover, the manuscripts were unable to be reviewed if it cannot be presented in a type of full-text. A fracture-dislocation was diagnosed when radiographs demonstrated ankle fractures with the presence of separation of the talus relative to the tibia on either the anteroposterior or lateral view. The search was performed by each reviewer independently with any disagreements in article eligibility resolved by the consensus discussion among all authors. Moreover, studies on the ankle fracture-dislocations that were written in English and presented as full-text were included in this review. Studies that were not written in English, not related to the topic of ankle fracture-dislocations, pure ankle dislocations, included only specific types of ankle fracture-dislocations, such as the “Logsplitter” injury (7), and lacked population characteristics were also excluded.

### Data extraction and study quality assessment

All data were collected by the two reviewers according to the uniform criteria. We documented the first author, title, published journal, year, type of study, design, and level of evidence of each study. The demographic data were recorded and extracted, including the groups, sample size, gender and age. The detailed data includes time from injury to surgery, operative time, fracture classification, surgical technique, clinical outcomes and postoperative complications. Moreover, the postoperative complications mainly included infection, posttraumatic arthritis, delayed union, nonunion, malunion and so on. The level of evidence was evaluated based on the guidelines of the Oxford Centre for Evidence-Based Medicine (8).

### Statistical analysis

The overall data in this current review were exhibited as the count (percentage) or mean ± standard deviation (SD). The pooled means were calculated for the mean age, time from injury to surgery and length of follow-up.

## Results

**Figure 1:**
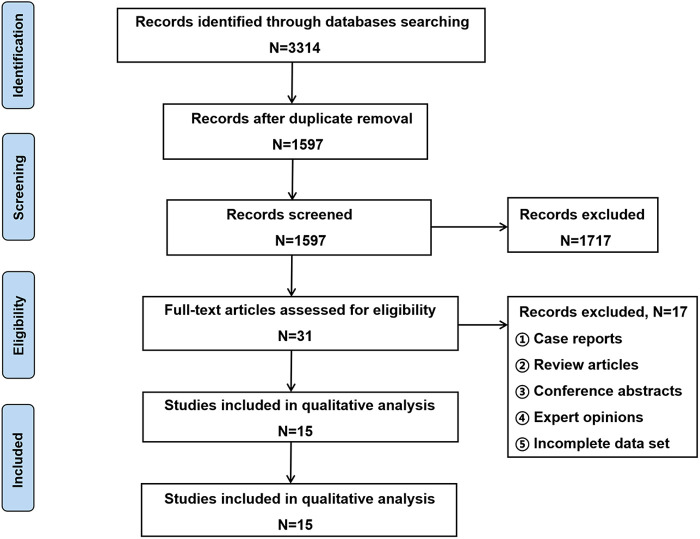
Preferred reporting items for systematic reviews.

The results of search strategy and study selection criteria are shown in Figure 1, and a total of 15 studies were included in this study (3–6, 9–19). This study was reported on the basis of the Preferred Reporting Items for Systematic Reviews and Meta-Analyses (PRISMA) statement.

### Key information

A total of 1,089 patients were enrolled in 15 included studies. As shown in Table 1, eight of these studies focused on the complications and functional outcomes after the surgery of ankle fracture-dislocations. Seven studies focused on comparing different treatment approaches for ankle fracture-dislocations. Two of seven studies compared the efficacy of splinting versus temporary external fixation. Moreover, only one study was prospective, while the remaining were retrospective. Generally, the patients with ankle fracture-dislocations had worse functional outcomes compared to those without dislocations, but the postoperative complications were similar. In the initial course of treatment, the block in closed reduction was comparable to conscious sedation, and the temporary external fixation and two-stage surgery were recommended for the treatment of ankle fracture-dislocations.

**Table 1:** The key information of the 15 included studies.

Author, country	Year	Aim	Sample size	Design	Important findings	Theme
Johnson, UK	1988	To evaluate the complications after the treatment of ankle fracture-dislocations leaving the deltoid ligament unrepaired.	30	Retrospective cohort study	Leaving the deltoid ligament unrepaired in ankle fracture-dislocations results in prolonged pain and disability.	Functional outcomes
Godsiff, UK	1993	To compare the outcome of early motion and immediate plaster splintage after surgery of ankle fracture-dislocation.	47	Retrospective observational study	Immediate plaster splintage had a shorter hospital stay and less swelling than the early movement group, early movement was not recommended.	Early motion, outcome
Brian, USA	2008	To compare the effect of intra-articular block and conscious sedation for closed reduction of an ankle fracture-dislocation.	42	Prospective randomized trial	An intra-articular block provides a similar degree of analgesia compared to conscious sedation for closed reduction of ankle fracture-dislocation.	Initial treatment
Ye, China	2011	To evaluate the effect of bioabsorbable screws combined with an external fixator for open ankle fracture-dislocations.	16	Retrospective observational study	Bioabsorbable implants combined with an external fixator could be effective in the treatment of ankle fracture-dislocations.	Open fracture, Initial treatment
Jeffrey, USA	2012	To develop a stepwise approach for the treatment of ankle fracture-dislocations.	40	Retrospective observational study	An algorithm for initial management was developed and the bivalved below-the-knee fiberglass cast is recommended.	Initial treatment
Peter, USA	2016	To evaluate the effect of dislocations on functional outcomes in SER IV ankle fracture patients.	108	Retrospective cohort study	Ankle fracture-dislocation are associated with worse radiographic and functional outcomes.	Functional outcomes
Oguzhan, Turkey	2018	To evaluate the effect of a 2-stage surgery for the ankle fracture-dislocations with severe soft tissue injuries compared to a 1-stage surgery.	45	Retrospective cohort study	A 2-stage surgery can be performed safely in the patients with severe soft tissue injuries but did not affect the function scores, at a minimum of 12 months of the follow-up.	Surgery planning
Direk, USA	2019	To compare functional outcome after open reduction internal fixation in ankle fractures with and without dislocation.	118	Retrospective cohort study	Functional outcomes in fracture-dislocations were generally poorer in a median >3-year follow-up.	Functional Outcomes
Richard, USA	2019	To compare the effect of splinting and external fixation in the treatment of ankle fracture-dislocations.	56	Retrospective cohort study	Splinting was associated with an increased risk of complications when compared to an external fixator.	Initial treatment
Mustafa, Turkey	2020	To determine the frequency of complications after surgery and the relationship between the trauma mechanism and these comorbidities.8	38	Retrospective observational study	Functional outcomes were found to be worse in patients with open ankle fractures and the rate of arthrosis increased with age.	Complications, outcome
Yüksel, Turkey	2020	To investigate differences in injury mechanisms of ankle fracture-dislocations in respect of functional outcomes and complications.	285	Retrospective cohort study	Ankle fracture-dislocations was not seen to worsen functional results but arthrosis and ankle fracture-dislocations were determined more often in these patients.	Functional outcomes, Injury mechanisms
Mehmet, Turkey	2021	To compare the effect of splinting and external fixation in the initial treatment of ankle fracture-dislocations.	117	Retrospective cohort study	The risk of potential complications can be reduced with the use of an external fixator.	Initial treatment
Case, USA	2021	To examine the incidence of surgical site complications associated with open pronation-abduction ankle fracture-dislocations.	48	Retrospective observational study	Low risk of surgical site complications was associated with appropriate surgical debridement, early stabilization, and primary wound closure,	Open fracture, Comorbidities,
Loïc, Senegal	2021	To determine the incidence of osteoarthritis after the treatment of ankle fracture-dislocations in a resource-limited setting.	52	Retrospective cohort study	Ankle fracture-dislocations are associated with high rate of early posttraumatic ankle osteoarthritis.	Long-term complications
Stephen, USA	2021	To compare short-term functional outcomes in PER IV ankle fractures with and without dislocation.	47	Retrospective cohort study	SER IV fracture-dislocations had higher rates of malreduction and poorer functional outcomes than PER IV fractures with no dislocation.	Functional outcomes

### Population characteristics

As exhibited in the Table 2, only one study was a multi-center study, while the rest were single-center studies. The mean age of all patients was 44.84 years. Males and females were equally represented, accounting for 49.4% and 50.6% of the total number, respectively. Six studies reported the cause of injury, with the highest percentage of high-energy injuries (21.3%), followed by traffic accidents (17.9%).

**Table 2:** Demographics of the 15 included studies.

Author, country	Year	Patients	LoE	Gender, *n* (Male%)	Age, years	Types of center research	Causes of injury, *n* (%)
Johnson, UK	1988	30	IV	N/A	36.0	Single	N/A
Godsiff, UK	1993	47	IV	28/19 (59.5)	45.0	Single	N/A
Brian, USA	2008	42	I	16/26 (38.1)	45.5	Single	N/A
Ye, China	2011	16	IV	13/3 (81.3)	37.0	Single	Traffic accident, 10 (62.5) Fall from height, 3(18.75) Crush, 3(18.75)
Jeffrey, USA	2012	40	IV	21/19 (52.5)	44.0	Single	N/A
Peter, USA	2016	108	III	35/73 (32)	N/A	Single	N/A
Oguzhan, Turkey	2018	45	III	19/26 (42.2)	48.8	Single	Traffic accident, 5(11.1) Fall from height, 2(4.4) Simple, 35(77.8) Sports injury,3(6.7)
Direk, USA	2019	118	III	50/68 (42.4)	46.6	Single	N/A
Richard, USA	2019	56	III	19/27 (33.9)	46.8/57.2	Multiple (2)	High energy, 13(23.2)
Mustafa, Turkey	2020	38	IV	25/13 (65.8)	33.9	Single	High energy, 26(68.4) Low energy, 12(31.6)
Yüksel, Turkey	2020	285	IV	155/130 (45.6)	44.7	Single	N/A
Mehmet, Turkey	2021	117	III	66/51 (56.4)	47.5	Single	N/A
Case, USA	2021	48	IV	15/32 (31.9)	53.4	Single	Traffic accident, 22(45.8) Fall from height, 9(18.8) Same level fall,15(31.3) Crush,2(4.2)
Loïc, Senegal	2021	52	IV	30/22 (57.7)	37.2	Single	N/A
Stephen, USA	2021	47	III	31/16 (66.0)	49.0	Single	High energy, 5(10.6) Fall, 32(68.1) Pedestrian struck, 10(21.3)

Note: N/A, Not applicable; LoE, level of evidence.

### Clinical characteristics

As shown in the Table 3, nine studies were grouped to compare. Fourteen studies described the type of fracture, with the highest percentage of SER fracture (43.8%) followed by pronation-external rotation (PER) fracture (13.0%). Moreover, two studies documented the direction of dislocation, with the vast majority of these being lateral dislocations (50.0%) and posterior dislocations (38.9%). The pooled mean time from injury to surgery was 7.2 (range 4.7 to 11.0) days. The most significant method of treating ankle fracture-dislocations was the open reduction and internal fixation (ORIF) and was supplemented by closed reduction and internal fixation (CRIF) and external fixator or splinting.

**Table 3:** Clinical characteristics of the 15 included studies.

Author, country	Year	Groups	Patients	Fracture type	Direction of dislocation	Time from injury to surgery(d)	Treatment
Johnson, UK	1988	N/A	30	N/A	N/A	N/A	ORIF + CRIF
Godsiff, UK	1993	Early movement group	27	Weber A:4(14.8) Weber B:15(55.6) Weber C:8(29.6)	N/A	N/A	ORIF + Early movement
		Plaster group	20	Weber A:2(10.0) Weber B:10(50.0) Weber C:8(40.0)			ORIF + Plaster
Brian, USA	2008	Intra-articular block group	21	SER, 13(61.9) PER, 8(38.1)	N/A	N/A	Closed Reduction
		Conscious sedation group	21	SER, 14(66.7) PER, 7(33.3)			
Ye, China	2011	N/A	16	Grade III open dislocated ankle fractures	N/A	6.0	ORIF + External fixator
Jeffrey, USA	2012	N/A	40	SER:35(87.5)	N/A	N/A	Initial management at the emergency clinic
				Other types:5(12.5)			
Peter, USA	2016	Fracture-dislocation group	35	SER IV	N/A	N/A	ORIF + External fixator
		No dislocation group	73				
Oguzhan, Turkey	2018	2-Stage Surgery group	20	SER,12(60.0) PER,6(30.0) SAD,1(5.0) PAD,1(5.0)	N/A	N/A	ORIF + External fixator
		1-Stage Surgery group	25	SER,14(56.0) PER,7(28.0) SAD,2(8.0) PAD,2(8.0)			ORIF
Direk, USA	2019	Fracture-dislocation group	33	Weber B:22(66.7) Weber C:11(33.3)	N/A	4.2–7	ORIF
		No dislocation group	85	Weber B:61(71.8) Weber C:24(28.2)		8.5–12	ORIF
Richard, USA	2019	External Fixation group,	28	SER,23(82.1) PER,5(17.9)	N/A	N/A	ORIF + External fixator
		Splint group	28	SER,28(100.0)			ORIF + Splint
Mustafa, Turkey	2020	N/A	38	SER,10(26.3) PER,15(39.5) SAD,4(10.5) PAD,9(23.7)	Anterior,5(13.2) Lateral,13(34.2) Medial,3(7.9)	4.7	ORIF
					Posterior,17(44.7)		
Yüksel, Turkey	2020	Fracture-dislocation group	88	SER,52(59.1) PER,22(25.0) SAD,1(1.1) PAD,13(14.8)	N/A	N/A	ORIF + CRIF
		No dislocation group	197	SER,155(78.7) PER,21(10.7) SAD,18(9.1) PAD,4(2.0)			
Mehmet, Turkey	2021	Fixator group	48	44B-2,2(4.2) 44B-3,34(70.8) 44C-2,12(25.0)	N/A	7.0	ORIF + External fixator
		Splint group	69	44B-2,4(5.8) 44B-3,44(63.8) 44C-2,21(30.4)		11.0	ORIF + Splint
Case, USA	2021	N/A	48	Open PAD	N/A	N/A	ORIF
Loïc, Senegal	2021	N/A	52	Bimalleolar,38(73.1) Trimalleolar,8(15.4)	Anterior,1(1.9) Lateral,32(61.5) Posterior-medial,1(1.9) Posterior,18(28.8)		Surgical/Conservative
				Unimalleolar,4(7.7) Other type,2(3.8)			
Stephen, USA	2021	Fracture-dislocation group	20	PER IV	N/A	N/A	ORIF
		No dislocation group	27				

Note: N/A, Not applicable; ORIF, open reduction and internal fixation; CRIF, close reduction and internal fixation.

### Clinical outcomes

As shown in Table 4, the pooled mean follow-up time was 21.7 (range 8.4 to 41.0) days. Twelve studies reported the postoperative complications, including the wound complications in 62 patients (6.5%), arthritis in 62 patients (6.5%), and delayed healing and nonhealing in 7 patients (0.7%) and 13 patients (1.4%), respectively. Of the 12 studies that reported functional evaluation criterion, 3 studies used FAOS score, while 5 studies used AOFAS score as the functional evaluation score.

**Table 4:** Clinical outcomes of the 15 included studies.

Author, country	Year	Groups	Patients	Length of follow-up, month	Postoperative complications, *n* (%)				Evaluation criterion of functional outcomes
					Wound complication	Delayed union	Malunion	Posttraumatic arthritis	
Johnson, UK	1988	N/A	30	15.0	0	0	0	1 (3.3)	Abduction/external rotation tests
Godsiff, UK	1993	Early movement group	27	N/A					Scoring system detailed by Baird and Jackson
		Plaster group	20						
Brian, USA	2008	Intra-articular block group	21	N/A					Pain score
		Conscious sedation group	21						
Ye, China	2011	N/A	16	18.1	4 (25.0)	0	0	2 (12.5)	Scoring system detailed by Wiss
Jeffrey, USA	2012	N/A	40	N/A					N/A
Peter, USA	2016	Fracture-dislocation group	35	21.0	6 (17.1)	0	0	0	FAOS score
		No dislocation group	73		8 (11.0)	0	0	0	Range of motion
Oguzhan, Turkey	2018	2-Stage Surgery group	20	19.2	2 (10.0)	3 (15.0)	0	0	AOFAS score
		1-Stage Surgery group	25	21.7	2 (8.0)	2 (8.0)	0	0	Olerud-Molander ankle score
Direk, USA	2019	Fracture-dislocation group	33	41.0	1 (3.0)	0	0	1 (3.0)	FAOS score
		No dislocation group	85	39.0	5 (5.9)	0	1 (1.2)	0	
Richard, USA	2019	External Fixation group	28	8.5	0	0	0	0	N/A
		Splint group	28	8.4	5 (17.9)	0	0	0	
Mustafa, Turkey	2020	N/A	38	33.6	0	0	0	16 (42.1)	AOFAS score
Yüksel, Turkey	2020	Fracture-dislocation group	88	38.4	1 (1.1)	0	2 (2.3)	7 (8.0)	AOFAS score
		No dislocation group	197		1 (0.5)	0	1 (0.5)	2 (1.0)	VAS score
Mehmet, Turkey	2021	Fixator group	48	16.0	3 (6.3)	0	0	0	AOFAS score
		Splint group	69	18.0	5 (7.2)	0	0	0	VAS score
Case, USA	2021	N/A	48	12.3	14 (29.2)	0	3 (6.3)	0	N/A
Loïc, Senegal	2021	N/A	52	27.2	0	2 (3.8)	6 (11.5)	19 (36.5)	AOFAS score
Stephen, USA	2021	Fracture-dislocation group	20	31.0	1 (5.0)	0	0	7 (35.0)	FAOS score
		No dislocation group	27	31.0	4(14.8)	0	0	5(18.5)	

Note: N/A, Not applicable; FAOS, Foot and Ankle Outcome Score; AOFAS, American Orthopedic Foot and Ankle Society Score; VAS, visual analogue scale.

## Discussion

Based on the key findings of our systematic review of ankle fracture-dislocations, most studies have focused on the treatment and functional outcomes. In order to explore the injury in depth, we decided to look at the anatomy as well as the pathological mechanisms of the ankle joint and further discuss the treatment as well as the prognosis by analyzing the pattern of injury. The purpose of this systematic review is to provide a more scientific and comprehensive understanding of ankle fracture-dislocations to guide treatment selection and subsequent related studies from the following aspects.

### Epidemiology

Ankle fractures are one of the most common types of lower-extremity fractures, accounting for approximately 10% of all fractures (1, 20–22). Dislocation referred to a complete discontinuity of the tibial talocrural joint, while subluxation was defined as a loss of coherence of the articular surfaces in contact with the articular cartilage with respect to the tibia on either the anteroposterior or lateral view **(**24). The proportion of ankle subluxations or dislocations is difficult to realistically reflect because many patients with ankle fracture-dislocations may have undergone closed reduction prior to radiographic evaluation. Several studies have shown that dislocation occurs in approximately 30%–50% of ankle fractures (3–5), and risk factors associated with ankle fracture-dislocation were also noted, including the old age, female, diabetes, and so on (5). Other than that, the probability of recurrence of ankle dislocation was minimal, compared to the high re-dislocation rate of shoulder and hip dislocations (23). Speaking further back to the dislocation, lateral, posterior and posterolateral were the top three subtypes of the ankle dislocations. Predictably, the type of ankle fracture most prone to dislocation is the triple ankle fracture, which accounts for nearly half of the patients. In addition, about 7% of patients were combined with the open ankle fracture-dislocations (24).

### Anatomy

The ankle joint is consisted of the talocrural joint and distal tibiofibular joint. Therein, the talocrural joint, is a modified hinged and uniaxial joint and the lateral surface of medial ankle is covered with cartilage as seen in an arthroscopic anatomical study (25). The talar body is wedge-shaped and is approximately 4–5 mm wider anteriorly than posteriorly (22), and the width between medial and lateral ankles is greater anteriorly than posteriorly, which allows the trochlea to closely match the malleoli during plantar dorsiflexion and flexion (26). The tight connection of three bony structures forms the cornerstone of ankle stability.

**Figure 2:**
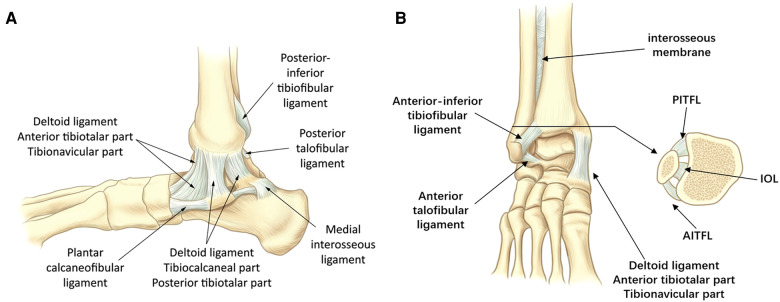
The periprosthetic ligament of the ankle joint complex. (**A**) Medial view. (**B**) Anterior view. Note: AITFL, anterior tibiofibular ligament; IOL, interosseous ligament; PITFL, Posterior tibiofibular ligament.

Moreover, the ankle joint is primarily supported by three groups of ligaments (Figure 2), including the lateral ligament, medial deltoid ligament, and the ligaments of tibiofibular syndesmosis. The lateral collateral ligament is consisted of anterior talofibular ligament (ATFL), calcaneofibular ligament (CFL), and posterior talofibular ligament (PTFL). The ATFL is the most vulnerable of ankle ligaments and has a role in limiting the anterior displacement of talus and plantar flexion (27). The PTFL is a multi-fascicular ligament that primarily limits the external rotation of talus and is tense during the process of plantar dorsiflexion (28, 29). Moreover, medial deltoid ligament is often considered to be the strongest of the peri-ankle ligaments. The deltoid ligament limits the external rotation of talus and excessive valgus of ankle joint. It is susceptible to injury during the excessive anterior external rotation or posterior external rotation of the ankle (30).

The tibiofibular syndesmosis is generally composed of the anterior inferior tibiofibular ligament (AITFL), posterior inferior tibiofibular ligament (PITFL) and interosseous tibiofibular ligament (IOL). Functionally speaking, the role of the AITFL is to tighten the fibula to the tibia and limit the excessive motion of fibula and external rotation of talus (32, 33). A cadaveric study showed that cutting only the AITFL resulted in almost three times the distance of talus displacement compared to cutting only the PITFL. This result suggests that the AITFL appears to be more important for ankle stability (34).

The PITFL is generally considered to have two parts (a superficial and a deep component). Importantly, partial analyses indicate that transverse ligament is an important structure independent of the posterior inferior tibiofibular ligament (31). Its fibers often reach the medial ankle and form a posterior labrum deepening the articular surface of the distal tibia, and this structure can limit posterior displacement of the talus (35, 36).

The IOL is considered to be the distal continuation of interosseous membrane of tibiofibular joint, which mainly inhibits the lateral translation of fibula (37, 38). Several mechanical analyses have shown that the IOL is stronger than the AITFL (38, 39), and the injury to IOL is often accompanied with the injury to AITFL (32).

### Pathophysiology

In terms of the injury pattern of ankle fracture-dislocations, strong violence causes the fracture and ligament damage and eventually results in the loss of alignment of the articular surfaces.

**Figure 3:**
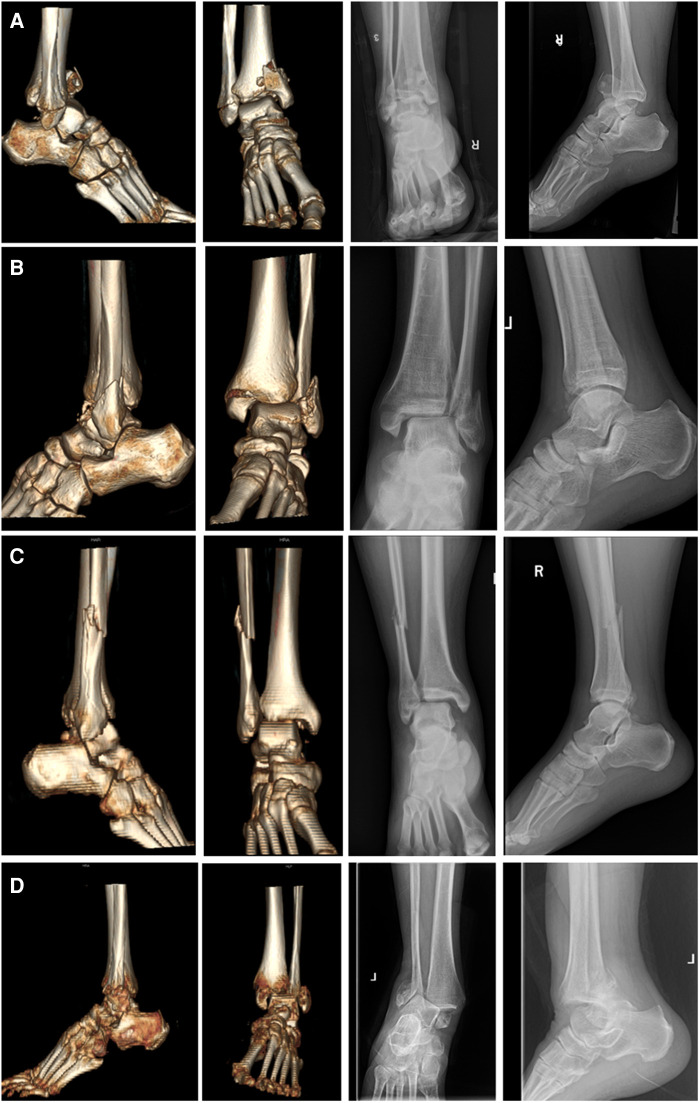
The CT reconstructions and radiographs of ankle fracture-dislocations with four different injury mechanisms according to the Lauge-Hansen's classification. (**A**) Supination-adduction; (**B**) Supination-external rotation; (**C**) Pronation abduction; (**D**) Pronation-external rotation.

The Lauge-Hansen's classification is the basis of ankle fracture mechanism and four types of ankle fracture-dislocations according to classification are exhibited in Figure 3 (40). The most common type of Lauge-Hansen classification is the SER ankle fracture, which accounts for approximately 60%–85% of all ankle fractures (41, 42). One study showed that 25% of SER ankle fractures were combined with dislocation (6). Further, partial studies showed a higher percentage of SER III/IV fractures in the ankle fracture-dislocations group (3, 5, 43). Based on the sequence of violence transmission, it can be further hypothesized that SER IV ankle fractures are more likely to be accompanied with syndesmotic injury. Moreover, this injury pattern is also more likely to result in the occurrence of dislocation. The suggested mechanisms of syndesmotic injury include the external rotation of foot, the eversion of talus within the ankle mortise and excessive dorsiflexion (44, 45). In these patterns of injury, the distal fibula is pushed away from the distal tibia and causes the ankle mortise to widen. Hence, the severe injuries to syndesmosis often lead to the dislocation of ankle (46). In this case, with the direction of violence, the talus would dislocate laterally and posterior-laterally, followed by the dislocation to posterior side.

In addition, it is clear that the AITFL will be the first to rupture. Since PITFL is a relatively strong ligament, its injury often takes the form of an avulsion posterior ankle fracture. Its deeper layer, the transverse ligament, forms a posterior labrum that limits the posterior displacement of talus, this results in a relatively rare posterior dislocation of talus. When the talus is extremely externally rotated, the injury to PTFL can occur and are essentially only seen in cases of ankle dislocation (47). The SER IV ankle fractures include either a rupture of the deltoid ligament or a medial ankle fracture, and a distinction needs to be made between these two different injuries when a dislocation occurs. Generally, the dislocation occurs only when there is a rupture of the deep portion of deltoid ligament (13).

As one of the most severely injured and intricate types of ankle fractures, a recent study has confirmed that trimalleolar fractures have a higher risk of dislocation (43). Biomechanical studies of trimalleolar fractures have found that contact stresses are concentrated on the articular cartilage (48). In addition to instability of the medial structures, ankle fracture-dislocations are also associated with larger posterior malleolar fragment (49). Posterior ankle malformations can lead to posterior lateral subluxation of the talus. In addition, the certain AITFL and PITFL injury and the underlying IOL injury in trimalleolar fractures contribute to instability of the syndesmosis. By reason of the foregoing, trimalleolar fractures are usually combined with injuries to the medial deltoid ligament as well as the syndesmosis, and the chances of dislocation are greatly increased with the addition of posterior ankle fractures.

The PER fractures are severe ankle injuries and account for approximately 14%–22% of all ankle fractures (42, 50). A recent study showed that about 41% of PER ankle fractures were dislocated. Unlike SER ankle fractures, the PER ankle fractures are first injured medially and the lateral fracture line is higher than the ankle plane. The injury of medial deltoid ligament will result in the talar instability (51, 52). A mechanical model study showed that when the deep deltoid ligament is ruptured, even if the fibula and syndesmosis were stable, the degree of external rotation in plantar flexion increases and the ankle dislocates at 20° to 30° plantar flexion, which also means that the talus will dislocate laterally. The most common types of dislocations in PER ankle fractures are the lateral dislocations (53). Noteworthily, when the syndesmosis injury is combined with deltoid ligament injury, the unstable talus rotates anterolaterally resulting in a decrease in tibiofibular joint contact area and an increase in intra-articular pressure, resulting in the subsequent dislocation (54). In general, the deltoid ligament, especially the deep deltoid ligament, is particularly important in the mechanism of dislocation in PER ankle fractures.

The other two uncommon types reveal relatively significant differences in dislocation rates. The probability of dislocation is 76% for the pronation-adduction (PAD) ankle fracture versus 5% for supination-adduction (SAD) ankle fracture (6). The greater violence of a PAD ankle fracture first damages the deltoid ligament, leading to the medial instability of ankle while the lateral structures of SAD ankle fracture are damaged first. Based on the previous analyses of mechanism, this is a situation in which the PAD ankle fractures are more likely to dislocate laterally. In contrast, the SAD ankle fractures can dislocate medially, but the probability of this occurring is relatively small.

### Clinical classification

The ankle fracture-dislocations are consisted of the fractures with total dislocation and subluxation. According to the relationship between direction of talus displacement and ankle mortise, ankle dislocations are classified into five types, including the anterior, posterior, medial, lateral, superior or the combination of these directions. The anterior ankle dislocation is generally where the foot remains stable, the ankle is forced into dorsiflexion, and there is a backward force on the lower leg. Due to the anterior dislocation of the talus, the dorsalis pedis artery may be damaged as a result (6, 55–57). The posterior ankle dislocation is the most common type of the dislocation and is often associated with injury to the tibiofibular syndesmosis and lateral malleolus fracture. There is also a greater risk of the injury to the posterior tibial nerve and accompanying vascular structures (58, 59). The medial and lateral ankle dislocations usually result from the eversion and inversion due to high intensity violence. The posterior medial dislocation often destroys the nervi peroneus superficialis and its related branches. The superior dislocations often occur in the falling injury when the talus is driven up into the mortise, and results in joint diastasis, disrupting the syndesmosis and allowing for talar dislocation. The superior dislocation can occur with or without the associated fractures (2, 60, 61). Regarding this, Ramasamy et al. (62) classified the complete ankle dislocation into two types. Type I is a dislocation without medial ankle fracture, and the type II is a dislocation with the medial ankle fracture. This typing strategy highlights the impact of injury to the medial ankle structures on the outcome of ankle fracture-dislocations.

### Treatment

#### Closed reduction and external fixation after reduction

Ankle fracture-dislocations may increase the risk of the injury to soft tissues surrounding ankle and overlying skin and consequently result in severe complications. The main indication for conducting a non-surgical closed reduction is ankle subluxation (63). Non-displaced fractures generally do not require closed reduction. It is best to obtain radiographs prior to closed reduction to understand the type and extent of the ankle injury (64). Generally, the reduction is usually accompanied with the appropriate analgesia and conscious sedation. Recently, the local hematoma block has also been recommended for the closed reduction of ankle fracture-dislocations. The hematoma block can provide a similar effect compared to the anesthesia without additional cardiovascular risk (15). Most of the time if neurovascular injury is clearly or highly suspected, the time of closed reduction should not be delayed due to x-ray. The primary goal of closed reduction is to reduce the impact of ankle fracture-dislocations on the skin and soft tissues.

External fixation is mainly the application of gypsum or splinting after the closed reduction. The ankle is usually immobilized in a neutral position to maintain the ankle stability and avoid contracture of Achilles tendon, and the care must be taken to avoid the skin compression when using the plaster fixation, especially in the elderly who can develop the severe skin ulcers as a result (65). In addition, compared to the temporary external fixators, a recent study found that in the patients with ankle fracture-dislocations who did not undergo emergency ORIF, splinting increased the risk of complications (such as re-dislocation and skin necrosis) (19). Hence, when a patient experiences the ankle fracture-dislocations, the extent of their ankle injury should be carefully evaluated and splinting should be used with caution.

#### Surgical timing

The surgical timing for ankle fractures is not yet definitive. Generally, the ankle will begin to swell continuously within 48 h after the fracture occurs, especially if the ankle is dislocated. Most surgeons are concerned about the skin complications associated with early surgery on an overly swollen ankle (66–68). Current German guidelines for ankle fractures recommend the surgery should be performed within 6–8 h, but these recommendations should also be revalidated in further prospective randomized studies. Several studies have indicated that the early surgery for ankle fractures can shorten the length of hospital stay (LHS), reduce the hospital costs, and increase the rate of anatomic reductions (69–73). A case-control study revealed that the effect of emergency surgery and elective surgery for ankle fracture-dislocations was roughly consistent, but the LHS of emergency surgery was shorter and the cost was less, which can relatively save the medical expenses of patients. In contrast, postoperative wound complications are a major concern for the surgeons. If the ankle joint is highly swollen and the skin develops the tension blisters, the risk of surgical incision infection and necrosis will increase due to the excessive tension. However, partial studies have indicated that the early surgery has lower infected wound complications and a better functional prognosis than delayed surgery (20, 66). Currently, there are still no related study focusing on the surgical timing for ankle fracture-dislocations, and further studies are still needed to support the idea of early surgery.

#### Surgical treatment

The purpose of ankle fracture surgery is to restore the fibula length and maintain the stable anatomical reduction. The treatment of open ankle fracture-dislocations focuses on avoiding the infection, reducing the rate of complications, promoting the bone healing and restoring the good function (74). The most common complications include deep infection (17%) and skin necrosis (18%) (75). An evidence-based study provides several guidelines for the surgical treatment of the open ankle fractures. In short, strong internal fixation should be given to restore the anatomy of the ankle fossa, and external fixation should be considered only if the soft tissue cannot cover the internal fixation. If the wound has no tension or needs to be opened for other purposes, the Grade I wound can be closed. Grade II wounds should wait for delayed closure or close after postoperative exclusion of infection. Grade III wounds should remain open and require postoperative skin grafting or flap treatment (76).

Ankle fracture-dislocations represent a more significant injury to the bony and soft-tissue structures of ankle joint. Thus, we need to focus on the treatment of periarticular ligament injuries in addition to ORIF routinely. Based on the previous analysis of the mechanism of injury, the ankle fracture-dislocations are usually combined with the syndesmotic injuries. Moreover, the instability of the syndesmotic complex may result in the long-term dysfunction and osteoarthritis (77). Although a prospective randomized study has revealed that the insertion of inferior tibiofibular screws is unnecessary in the SER-type ankle fractures (78). However, the current overwhelming opinion is that the insertion of syndesmosis screws is the gold standard when a syndesmotic injury has occurred (79, 80). The syndesmosis screws are typically inserted 2–3 cm above the articular surface and is angled approximately 20° anteromedially (81, 82). There is insufficient evidence for the early removal of syndesmosis screws and the sufficient time may be required to ensure the adequate recovery of syndesmotic complex (83–85). However, the prolonged insertion may result in the loss of repositioning or screw fracture. Hence, further studies are still needed to clarify the optimal insertion time and position. Besides, a variety of novel surgical instruments have been developed to replace the syndesmosis screws recently. Five meta-analyses have compared the efficacy of suture-button versus screws and confirmed the similar functional outcomes and postoperative complications in both groups (86–90). Another novel surgical instrument is the bioresorbable screws, which has the advantage of not requiring screw removal. Two latest meta-analyses suggested a higher complication rate of bioresorbable screws compared to syndesmosis screws, and the use of bioresorbable screws is not recommended (86, 90). In addition, dynamic fixation is another surgical device that can ensure an effective reduction. A study indicated that the dynamic fixation reduces the complications and improves clinical outcomes, with a lower risk of reoperation (91).

Judging by the mechanism of injury, the ankle fracture-dislocations are likely to be accompanied with the injury to medial deltoid ligament. It is worth being vigilant that 58.3% of acute ankle fractures were associated with the medial triangular ligament injury (92). Approximately 39.6% of ankle fracture-dislocations were associated with the rupture of deltoid ligament (93). Furthermore, the non-anatomic healing of deltoid ligament may result in the persistent medial pain and ankle instability, and even the risk of early arthritis. The most common surgical approach to repair the deltoid ligament is fixing the superficial deltoid ligament and the deep deltoid ligament on a suture anchor inserted in the medial ankle (94–97). Several retrospective studies did not recommend the additional deltoid ligament repair, since it can scar heal and eventually become a functional ligament (98–100). Other studies have indicated better outcomes in the deltoid repair group in terms of pain scores and functional scores during the follow-up (101–103). However, a randomized controlled trial reported that the deltoid ligament repair was not necessary when the fibular anatomy returned to normal. Although this trial had limitations such as no analysis of violence energy and no assessment of medial stability (104). In a recent Meta-analysis, the authors found that patients who repaired the deltoid ligament had better radiographic correction of the medial clear space and had relatively better pain scores (105). In summary, additional deltoid ligament repair surgery may be helpful in terms of repositioning results and pain scores in the early stages of recovery, but it also adds additional operative time and cost. A syndesmotic reduction could be much more significant than deltoid repair in restoring the mortise and keeping ankle stability.

### Efficacy and prognosis

With the increasing demand for functional movement in modern society, more and more orthopaedic surgeons are currently concerned about the functional outcomes of ankle fracture-dislocations. A study on the prognosis of ankle fracture-dislocation found that the functional outcomes were worse in the open ankle fracture-dislocations. Talar cartilage injury after dislocation tends to be prone to arthrosis, and its prevalence increases with age (18). However, in a retrospective study analyzing mechanism and functional outcomes of dislocations according to the Lauge-Hansen classification, the investigators found that functional outcomes did not worsen in the mid-term, but the patients with dislocations were more likely to develop the reflex sympathetic dystrophy syndrome (6). Moreover, another analysis showed no increased wound complications but worse functional outcomes in the dislocation group, except for the lower pain scores (5). These results suggest that more detailed and precise studies need to be further implemented in the future. In the patients with SER IV ankle fractures, the dislocation is associated with worse radiographic and functional outcomes (3). Other than that, the dislocations may result in higher rates of articular malreduction and worse functional outcomes in patients with PER IV ankle fractures due to higher energy damage (4).

## Conclusions

Ankle fracture-dislocations are mostly due to the high-energy violence and can be accompanied with varying amount of bone and soft tissue injury. Dislocation often leads to worse functional outcomes in ankle fractures. According to Lauge-Hansen's classification, the injury of the deep deltoid ligament is closely related to the injury mechanism of SER and PER ankle fractures. Conservative treatment is not recommended in the event of fracture-dislocation. In addition to conventional ORIF, syndesmotic fixation is also important while additional deltoid ligament repair is unnecessary. Collectively, the ankle fracture-dislocations still require a more comprehensive and clearer treatment guideline in the future, as well as the prognostic evaluation system.
